# Zooplankton Community Stability and Environmental Filtering in a Shallow Eutrophic Lake

**DOI:** 10.1002/ece3.73773

**Published:** 2026-05-31

**Authors:** Shihao Tang, Jianqiang Zhu, Zilong Nie, Jun R. Yang

**Affiliations:** ^1^ MARA Key Laboratory of Sustainable Crop Production in the Middle Reaches of the Yangtze River (Co‐Construction by Ministry and Province)/Hubei Key Laboratory of Waterlogging Disaster and Agricultural Use of Wetland, College of Agriculture Yangtze University Jingzhou China

**Keywords:** community stability, eutrophication, resilience, restoration, zooplankton

## Abstract

Zooplankton are key regulators of trophic interactions and nutrient cycling in shallow eutrophic lakes and are sensitive indicators of ecological responses to anthropogenic pressures. This study was conducted to understand ecosystem dynamics in Changhu Lake, a typical shallow eutrophic lake in China. We conducted monthly surveys from June 2023 to May 2024 in Changhu Lake. Our work involved identifying zooplankton species and analyzing their assemblages. We used redundancy analysis (RDA), a neutral community model, and structural equation modeling (SEM) to identify key environmental drivers and understand community assembly processes. We identified 102 zooplankton species. The assemblages showed marked spatiotemporal variations, with small‐bodied taxa dominating most seasons and sites, while large crustaceans remained scarce and peaked only locally in spring. Diversity peaked in autumn and declined sharply in summer. Compared to data from 2012 to 2013, total zooplankton abundance increased and small taxa became more dominant. RDA identified total phosphorus, chlorophyll *a*, ammonium nitrogen, and water temperature as key environmental drivers. Neutral community modeling indicated a higher migration rate in spring (*R* ^2^ = 0.610, Nm = 47) versus dispersal limitation in winter (*R* ^2^ = 191, Nm = 15). The community structure had a direct positive effect on ecosystem stability, whereas diversity had an indirect negative effect. The findings indicate a limited recovery of large‐bodied species despite ongoing restoration initiatives. This study provides mechanistic insights into community assembly and resilience, offering valuable guidance for the adaptive management of lakes facing intensifying anthropogenic pressures.

## Graphical abstract

Anthropogenic pressures led to zooplankton shift to small‐bodied dominance, not structural reorganization. Integrated management is crucial for long‐term resilience.

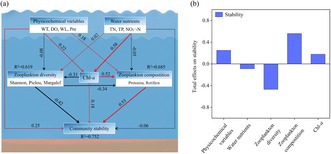

## 1 Introduction

Eutrophication remains a critical and persistent environmental challenge threatening global freshwater ecosystems. Shallow lakes, characterized by their limited depth, low water volume, and slow water exchange, are particularly vulnerable to nutrient accumulation and eutrophication (Pereira and Mulligan 2023; Qin et al. 2023). Excessive nitrogen and phosphorus inputs can trigger recurrent algal blooms, hypoxic events, and sharp declines in biodiversity, severely impairing water quality and ecosystem services (Abd. Razak and Sharip 2019). In recent decades, freshwater lake ecosystems have experienced accelerating degradation under intensifying anthropogenic pressures, manifested notably in the depletion of aquatic biological resources and the progressive erosion of biodiversity (Mutethya et al. 2024). Among these stressors, overfishing constitutes a particularly disruptive force, altering natural fish community structure and inducing cascading effects across trophic levels, ultimately destabilizing aquatic ecosystem processes (Lomartire et al. 2021).

Zooplankton are pivotal trophic intermediaries in aquatic food webs. They function as primary consumers of phytoplankton and essential prey for fish and macroinvertebrates. They play fundamental roles in nutrient cycling and energy transfer within freshwater ecosystems (Fahd et al. 2007; Cabral et al. 2020). However, under eutrophic conditions, phytoplankton overgrowth—particularly of cyanobacteria—can alter food availability both qualitatively and quantitatively, thereby affecting zooplankton population dynamics, species composition, and trophic interactions. These disruptions can reduce community stability and compromise overall ecosystem functioning (Chen et al. 2025). Therefore, zooplankton species diversity and community stability serve as central metrics for evaluating ecosystem health and resilience, especially in shallow eutrophic lakes (Alvarez et al. 2025). In such systems, zooplankton communities are shaped by a combination of abiotic and biotic drivers, including water level fluctuations, thermal regimes, nutrient enrichment, and predation pressures, resulting in pronounced spatiotemporal heterogeneity closely linked to water quality and interspecific competition (Yang et al. 2017; dos Santos et al. 2025).

The Yangtze River and its associated floodplain lakes form a complex river–lake ecotone that supports high aquatic biodiversity. Within this network, zooplankton, as a key functional group, significantly contribute to energy flow, biogeochemical cycling, and ecological stability, while also serving as reliable bioindicators of water quality and environmental change (Qi et al. 2025; Wang et al. 2025). Empirical studies have established close correlations between zooplankton biomass/density and total nitrogen, total phosphorus, and chlorophyll‐*a*. Species richness often exhibits a unimodal response to conductivity within the range of 400–1000 μS/cm (Yang et al. 2023). Furthermore, cyanobacterial dominance has been demonstrated to negatively impact zooplankton populations and reduce community diversity. The synergistic effects of nutrient loading and rising temperatures are recognized as key drivers of structural shifts in zooplankton communities (Zhao et al. 2021). Body size has also emerged as a sensitive bioindicator of environmental stress, with large‐bodied zooplankton taxa being more vulnerable to predation and physicochemical fluctuations (Wang et al. 2022). For example, studies in the Chaohu Basin have emphasized the role of spatiotemporal environmental heterogeneity in shaping functional group distributions (Wu et al. 2022), while long‐term monitoring in Lake Taihu has shown that eutrophication, coupled with climate change, has led to a gradual decline in rotifer abundance and a relative increase in copepods and cladocerans under more stable hydrological conditions (Zhou et al. 2020).

Changhu Lake, situated in Jingzhou City, Hubei Province, is a typical shallow eutrophic lake in the middle‐lower Yangtze River Basin. The lake has experienced sustained anthropogenic pressures—including agricultural runoff, urban expansion, and industrial discharge—resulting in chronic nutrient accumulation, persistent eutrophication, and elevated phytoplankton biomass. These conditions have driven ecological degradation and shifts in biotic communities. To mitigate the deterioration of watershed wetlands and facilitate the gradual restoration of the lake's natural ecosystem, the Changhu Lake Ecological Restoration Project was initiated (Wu et al. 2025). This initiative implemented key interventions, including the removal of aquaculture enclosures, stock enhancement, and the re‐establishment of submerged macrophytes. Owing to its pronounced seasonal hydrological variability and spatial heterogeneity, Changhu Lake provides an ideal natural laboratory for investigating zooplankton responses to multifactorial environmental stressors and for assessing spatiotemporal succession patterns and community assembly mechanisms.

Despite growing research interest in zooplankton ecology within eutrophic systems, integrated studies on the spatiotemporal patterns and assembly dynamics of zooplankton in Changhu Lake remain scarce. To address this knowledge gap, we conducted a year‐round field investigation from June 2023 to May 2024 to examine the ecological responses of the zooplankton community. The specific objectives were to: (1) characterize the seasonal and spatial distribution patterns of major zooplankton groups (protozoans, rotifers, cladocerans, and copepods); (2) evaluate the temporal dynamics of species diversity and community stability; (3) identify key environmental drivers shaping community structure and composition; and (4) apply neutral community models (NCM) and structural equation models (SEM) to elucidate the underlying assembly mechanisms and their influence on ecosystem stability.

By integrating community‐level metrics with environmental parameters and multivariate modeling, this study provides novel insights into the structure–function dynamics of zooplankton communities in shallow eutrophic lakes under anthropogenic stress. The findings offer a scientific basis for ecological health assessment and support the development of evidence‐based management strategies for lake ecosystem restoration.

## 2 Materials and Methods

### 2.1 Study Area and Sampling Sites

**FIGURE 1:**
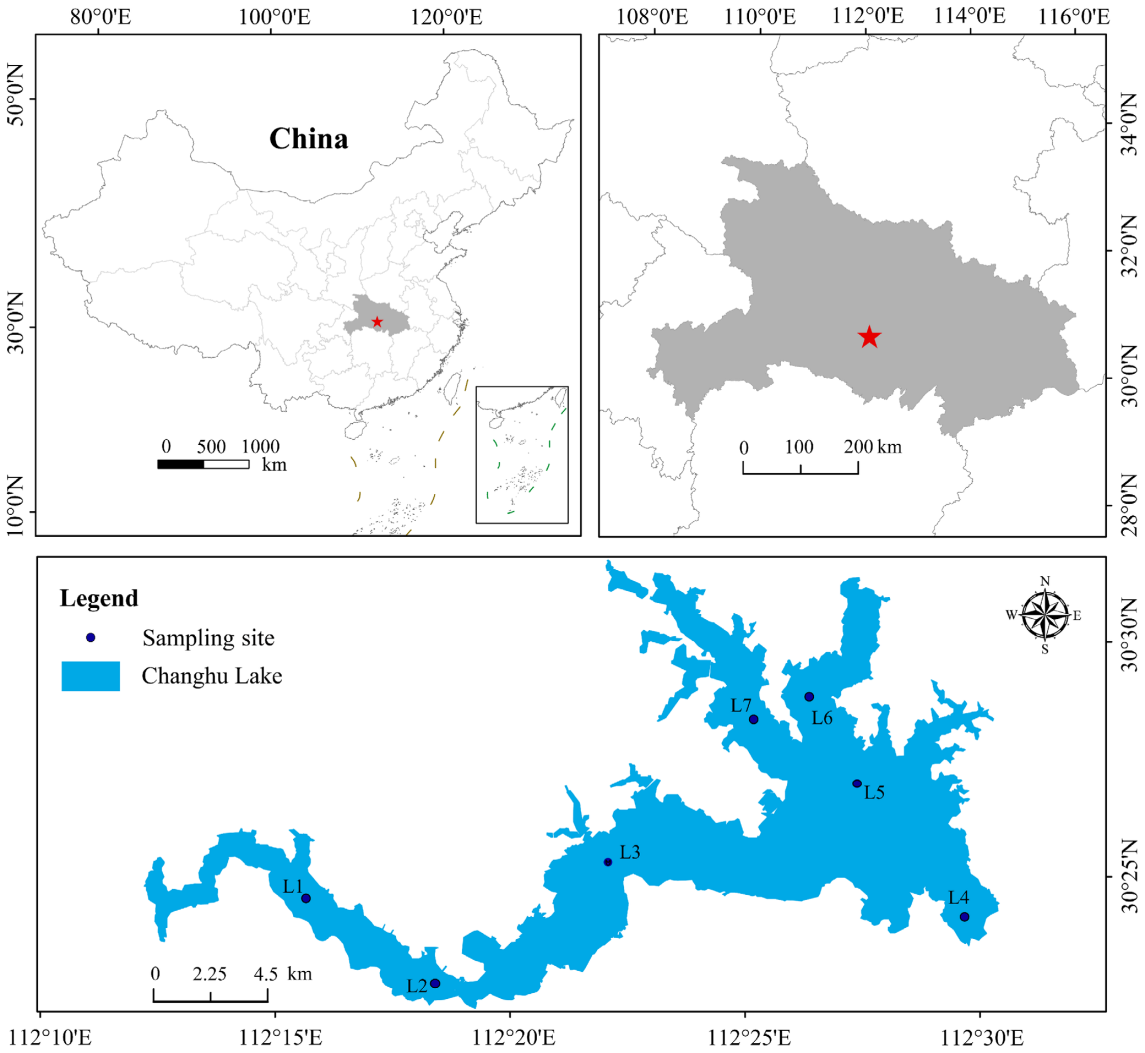
Location of Changhu Lake and sampling sites. The upper left panel shows the location of the lake (red star) within China. The upper right panel shows its position in Hubei Province. The lower panel details the distribution of the seven sampling sites (L1–L7).

Changhu Lake (30°22′–30°53′ N, 112°17′–112°50′ E), situated on the central Jianghan Plain in Hubei Province, central China, is the province's third‐largest freshwater lake (Figure 1). Spanning the administrative regions of Jingzhou, Jingmen, and Qianjiang, it exemplifies a typical shallow eutrophic lake system characterized by high nutrient loading and low water exchange capacity. The lake's surface area fluctuates seasonally between 122 and 150 km^2^, with a shoreline length of approximately 310 km. It has a maximum depth of 6.1 m, an average depth of 2.1 m, and a total water volume of 2.71 × 10^9^ m^3^. Located within a subtropical humid monsoon climate zone, the region experiences four distinct seasons with strong coupling between temperature and precipitation regimes. The annual mean temperature is approximately 16.5°C, and the average annual precipitation is about 1200 mm, predominantly occurring between May and September. Hydrologically, the lake is primarily recharged by surface runoff and direct precipitation, resulting in significant seasonal fluctuations in water level and hydrodynamic conditions.

Morphologically, Changhu Lake is elongated along an east–west axis, with a broader eastern basin and a narrower western section. The surrounding landscape has been substantially altered by anthropogenic infrastructure, including artificial embankments, drainage canals, and irrigation systems, forming a complex hydrographic network. The lake basin exhibits a distinct topographic gradient—higher in the northwest and lower in the southeast—and lies within a biogeographic transition zone between northern and southern China, contributing to its unique ecological and climatic characteristics. These attributes make Changhu Lake an ideal natural laboratory for studying ecosystem responses to multiple environmental stressors in shallow eutrophic freshwater systems.

Based on lake morphometry, hydrodynamic regimes, and ecological functional zoning, we systematically established seven representative sampling sites within Changhu Lake from June 2023 to May 2024 (Figure 1). These sites—Chenditou (L1), Songjiachong (L2), Longkouzhai (L3), Xikou (L4), Nanzui (L5), Guangpinggang (L6), and Hougang (L7)—were sampled monthly. Site selection was designed to capture the spatial heterogeneity of habitat types, hydrodynamic conditions, and trophic gradients, ensuring comprehensive representation of the lake's ecological variability.

### 2.2 Zooplankton Sampling and Processing

To characterize the aquatic environment, triplicate surface water samples (0–0.5 m depth) were collected monthly at each site for physicochemical analysis. All laboratory analyses were completed within 24 h of sample collection. Nine key water quality parameters were quantified: dissolved oxygen (DO), water temperature (WT), nitrate nitrogen (NO_3_^−^‐N), ammonium nitrogen (NH_4_^+^‐N), total nitrogen (TN), total dissolved nitrogen (TDN), total phosphorus (TP), total dissolved phosphorus (TDP), and chlorophyll *a* (Chl‐*a*). All analyses strictly followed national standardized protocols (Wei 2002). Each parameter was assayed in triplicate to ensure precision; mean values are reported, with measurement error controlled within 5%.

Qualitative zooplankton samples were collected using a No. 13 plankton net (64 μm mesh) by repeatedly towing just below the water surface along an “∞”‐shaped transect. Concentrated samples were preserved in 50 mL specimen bottles with 4% formaldehyde for taxonomic identification. Quantitative sampling employed a 5‐L water sampler to collect duplicate integrated water samples from the epilimnion, metalimnion, and hypolimnion. These replicate samples were homogenized and concentrated through a No. 25 plankton net (40 μm mesh) to a final volume of 100 mL, then fixed with 4% formaldehyde for quantitative analysis of Cladocera and Copepoda. Protozoa and Rotifera quantitative samples were derived from subsamples of the phytoplankton quantitative collection. For enumeration, 0.1 mL subsamples were pipetted into 0.1 mL counting chambers and analyzed in duplicate to minimize counting error (constrained within 15%). Quantitative enumeration of Cladocera and Copepoda was performed using 1 mL counting chambers, following procedures consistent with those for Protozoa and Rotifera (Kâ et al. 2012; Ahmed et al. 2025; Guo, Wang, et al. 2025).

### 2.3 Data Processing and Analysis

Zooplankton abundance was quantified following established protocols (The Ministry of Agriculture of the People's Republic of China 2011).

(1) $$N=\left(V_{s}\cdot n\right)/\left(V\cdot V_{a}\right)$$

where *N* represents the zooplankton abundance (ind./L), *V* denotes the volume of the collected water sample, *V*_s_ is the volume of the concentrated sedimented sample, *V*_*a*_ indicates the volume of the subsample used for counting, and *n* is the number of individuals counted.

Zooplankton biomass was estimated by converting individual abundance to wet weight, utilizing species‐specific mean individual weights (The Ministry of Agriculture of the People's Republic of China 2011).

Based on species abundance, Pielou's evenness index (*J*), Margalef's richness index (*D*), and Shannon‐Wiener diversity index (*H′*) were calculated for the zooplankton community. The water quality status was classified into five categories, ranging from “Very Poor” to “Excellent”, based on these indices (Table S1). The formulas for the indices are:

(2) $$J=H^{\prime }/\log_{2}S$$

(3) $$D=\left(S-1\right)/\ln N$$

(4) $$H^{\prime }=-\sum_{i=1}^{S}P_{i}\ln P_{i}$$

where *S* represents the total number of species, *N* is the total abundance of all species in the community, *P*_*i*_ denotes the proportion of individuals of species *i* relative to the total individuals, *n*_*i*_ is the abundance of species *i*, and *f*_*i*_ represents the frequency of occurrence of species *i* across sampling sites.

Community turnover and temporal stability (reflecting fluctuations in species biomass over time) were assessed. The Bray‐Curtis dissimilarity index was used to quantify zooplankton community turnover between sampling occasions (Ptacnik et al. 2008; Filstrup et al. 2014). The formula is:

(5) $$\mathrm{BC}=\frac{\sum_{i=1}^{n}\;\left|y_{i1}-y_{i2}\right|}{\sum_{i=1}^{n}\;\left|y_{i1}+y_{i2}\right|}$$

where *BC* is the community turnover value, *y*_*i1*_ is the first measured biomass of species *i* in the community (mg/L), *y*_*i2*_ is the second measured biomass of species *i* in the community (mg/L), and *n* is the total number of species identified in the two measurements.

Preliminary data processing was performed using Microsoft Excel. Community relative abundance and biomass visualizations were generated using Origin 2021. One‐way analysis of variance (ANOVA) was conducted in SPSS 27. Significant differences in diversity indices and community parameters among groups were assessed using least significant difference (LSD) and Duncan's multiple range tests. Spatial and temporal variations in zooplankton density and *α*‐diversity metrics were analyzed statistically. To elucidate the relationships between zooplankton community composition and environmental gradients, redundancy analysis (RDA) was employed. Additionally, a SEM was constructed to examine the direct and indirect effects of environmental factors (e.g., physiochemical variables and nutrients) on zooplankton diversity and community stability. The model was implemented in Amos 26.0 using maximum likelihood estimation (MLE) to compute standardized path coefficients. Only statistically significant pathways (*p* < 0.05) were retained in the final model, with path diagrams illustrating the causal relationships among variables (Guo, Li, et al. 2025).

To assess the assembly mechanisms of the zooplankton community, we applied the neutral community model (NCM), which is grounded in Hubbell's neutral theory (Hubbell 1997, 2001). This framework assumes functional equivalence among species, attributing community dynamics to stochastic processes, primarily ecological drift and demographic stochasticity (i.e., random variation in birth, death, and reproduction events). In this study, we fitted the cumulative abundance‐frequency distributions of zooplankton operational taxonomic units (OTUs) to the Beta distribution derived from Marquet et al. (2017). Specifically, the local community neutral model was fitted to zooplankton OTU abundances (pooled across all seven sites) for each season using maximum likelihood estimation. To assess model fit beyond the traditional R^2^ measure, we classified OTUs based on whether their observed occurrence frequencies fell within, above, or below the 95% confidence intervals of the model prediction. Therefore, we used the outlier proportion (the sum of below and above) as the indicator of model fit, complementing the goodness‐of‐fit statistic (*R* ^2^), defined as the square of Pearson's correlation coefficient between the model's mean trend and the observed data points. The relationship between the nonlinear least square fitting frequency and relative abundance was used to estimate the migration rate (Nm) and its confidence interval. By comparing empirical data against NCM predictions—with particular attention to the pattern of outliers—we quantified the relative contributions of stochastic (demographic stochasticity and drift) versus deterministic (environmental filtering) processes in shaping community composition. All model fitting and subsequent analyses were conducted using the Hmisc package in R Studio (version 4.2.2).

## 3 Results

### 3.1 Environmental Parameters

**FIGURE 2:**
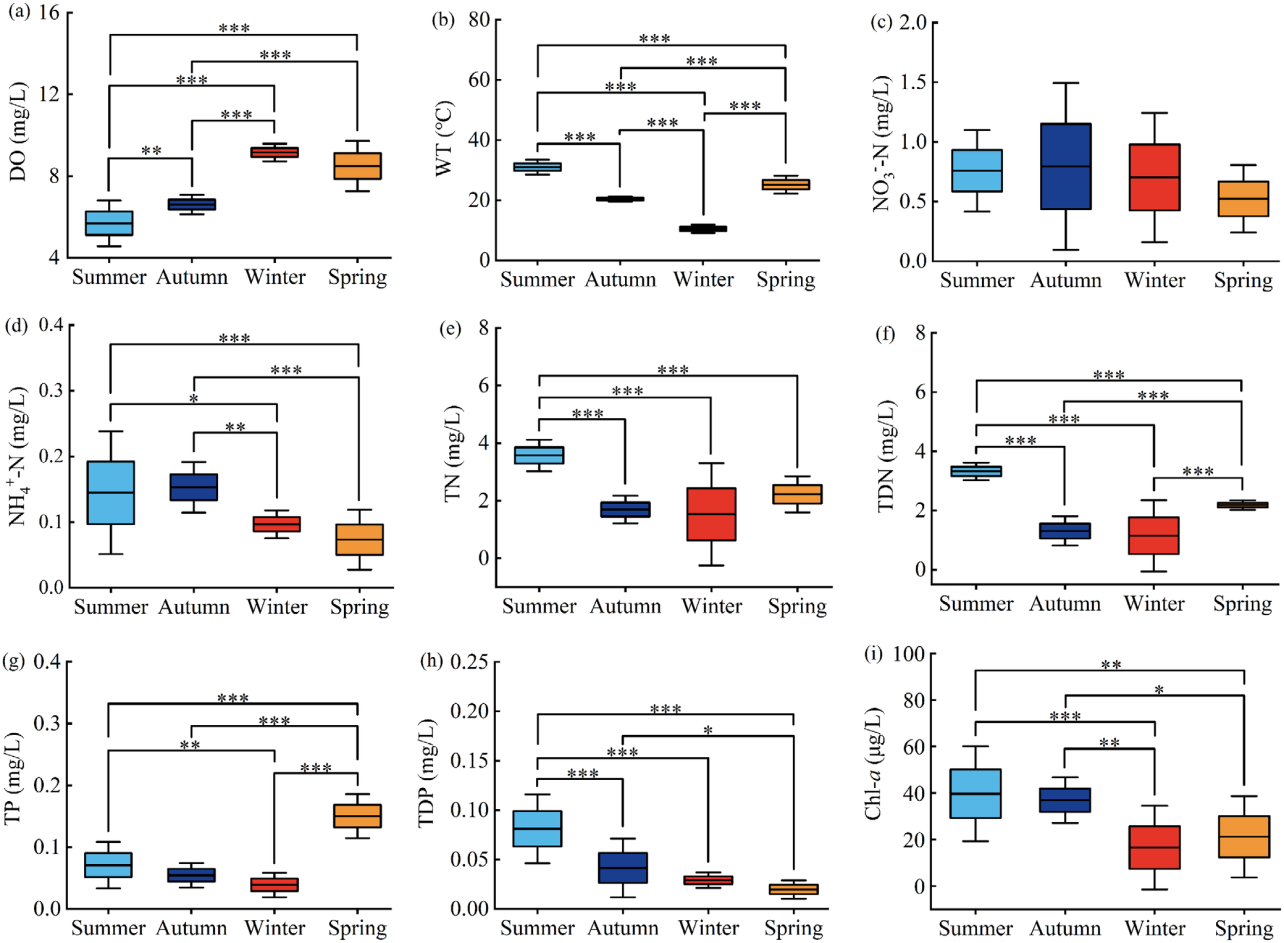
Seasonal dynamics of environmental factors in Changhu Lake. (a) Dissolved oxygen (DO), (b) Water temperature (WT), (c) Nitrate nitrogen (NO_3_^−^‐N), (d) Ammonium nitrogen (NH_4_^+^‐N), (e) Total nitrogen (TN), (f) Total dissolved nitrogen (TDN), (g) Total phosphorus (TP), (h) Total dissolved phosphorus (TDP), and (i) Chlorophyll‐*a* (Chl‐*a*). Asterisks indicate significant differences between seasons. *p < 0.05, **p < 0.01, ***p < 0.001.

All measured physicochemical parameters in Changhu Lake exhibited significant seasonal variability throughout the study (Figure 2). DO concentrations peaked in winter (9.2 mg/L) and reached their lowest level in summer (4.7 mg/L), with intermediate values recorded in autumn and spring. WT showed a clear seasonal cycle, highest in summer (33°C), decreasing from approximately 19.5°C in autumn to 9.3°C in winter, and rising again to around 27°C in spring. NO_3_^−^‐N concentrations remained relatively stable across seasons, while NH_4_^+^‐N was significantly elevated in summer and autumn, showing highly significant differences compared to winter and spring (*p* < 0.001). Both TN and TDN reached their annual maxima in summer (approximately 4.05 mg/L and 3.58 mg/L, respectively), followed by substantial declines in winter and spring (*p* < 0.001). TP and TDP exhibited similar seasonal trends, with concentrations significantly higher in summer and spring than in autumn and winter (*p* < 0.001). Chl‐*a* concentrations peaked in summer and autumn, significantly exceeding those in spring and winter (*p* < 0.001).

Precipitation and water level in Changhu Lake exhibited pronounced seasonal variability during the study period (Figure S1). Precipitation peaked in summer at 221 mm (Figure S1a), declined sharply to 29.5 mm in winter, and partially recovered to 89.5–121.5 mm in spring. Correspondingly, water level closely tracked precipitation patterns (Figure S1b), reaching a maximum of 31.33 m in autumn, decreasing to 30.39 m in winter, and showing a slight rebound to 30.41 m in spring.

### 3.2 Zooplankton Community Structure

#### 3.2.1 Species Richness

**FIGURE 3:**
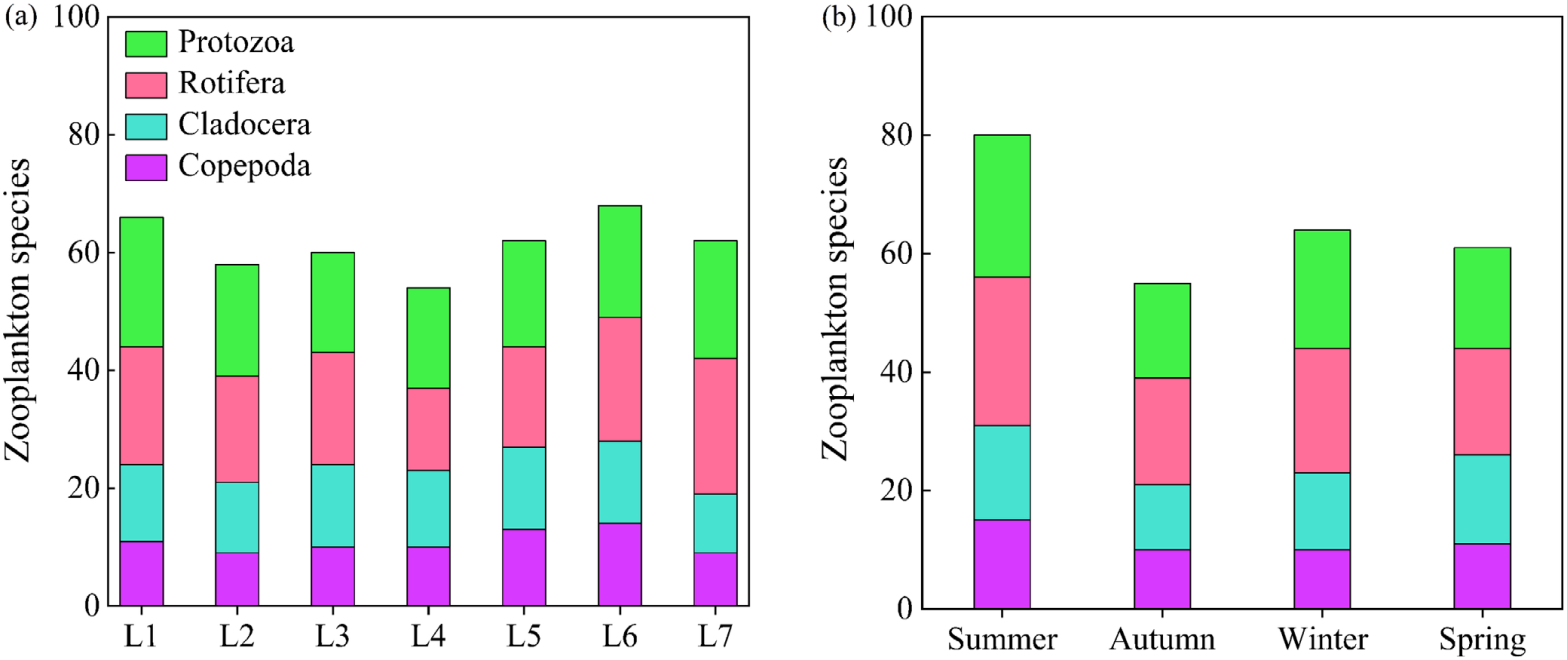
Spatial (a) and seasonal (b) variations of zooplankton community composition in Changhu Lake.

Zooplankton species richness showed little variation among sites or across seasons (Figure 3). Spatially, site L4 exhibited the lowest richness (54 taxa), while sites L3, L5, and L7 supported higher richness (60, 62, and 62 taxa, respectively; Figure 3a). Protozoans were a dominant component across all sites, with particularly high contributions at L1 (33%), L2 (33%), and L7 (32%). Rotifers were ubiquitous, with higher richness at L2 (31%), L3 (32%), and L7 (37%). In contrast, the larger‐bodied cladocerans and copepods showed lower species richness, with sporadic but notable presence mainly at L4 (43%), L5 (44%), and L6 (42%).

Seasonally, species richness peaked in summer (80 species), followed by winter (64), spring (61), and autumn (55; Figure 3b). Across seasons, protozoans and rotifers collectively accounted for over 58% of total richness, forming the predominant taxonomic groups. Overall, protozoans and rotifers consistently dominate across both spatial and temporal scales, exerting a pivotal influence on community structure and dynamics.

#### 3.2.2 Abundance

**FIGURE 4:**
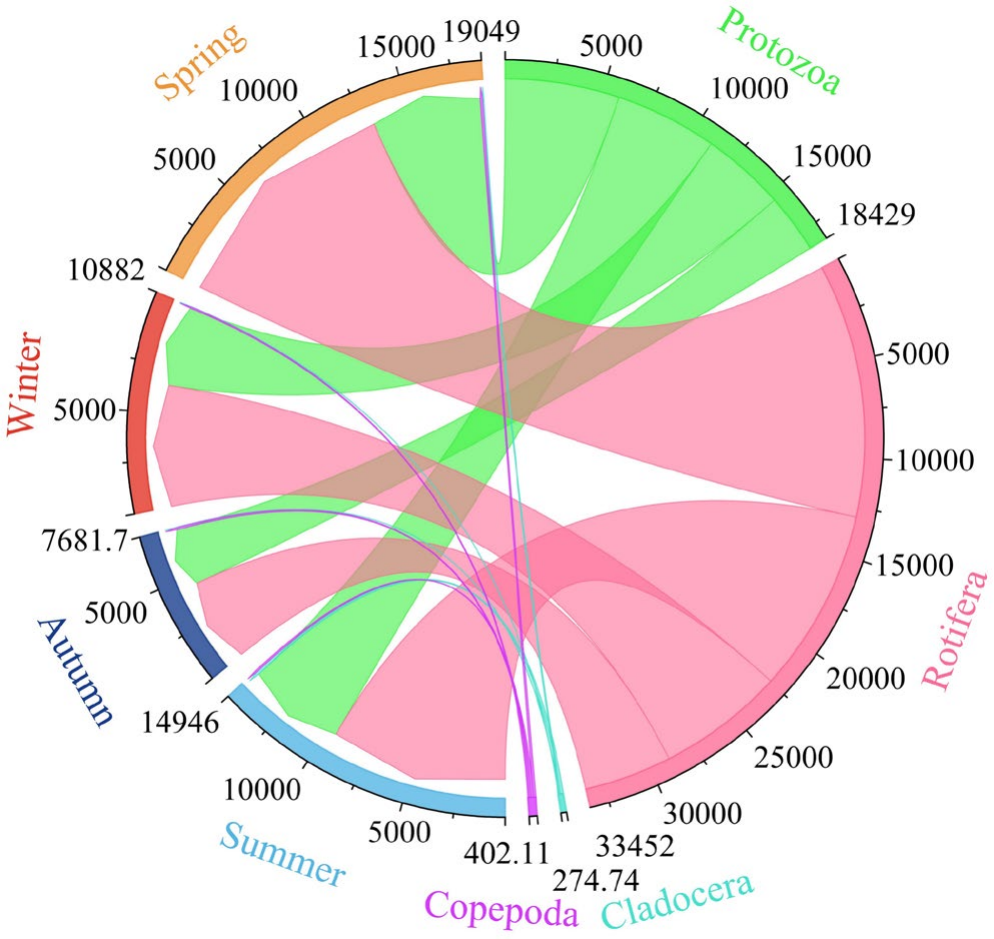
Seasonal variations of zooplankton abundance in Changhu Lake. The width of connections represents the abundance of zooplankton.

Zooplankton abundance in Changhu Lake exhibited significant seasonal variations (Figure 4). In summer, the community was overwhelmingly dominated by rotifers (peak density: 9452.38 ind./L), while protozoans, cladocerans, and copepods were less abundant. Total abundance declined to an annual minimum in autumn (7681.65 ind./L), with rotifers (4500 ind./L) and protozoans (3047.61 ind./L) as the dominant taxa. Abundance rebounded in winter (10,881.65 ind./L), with rotifers (6500 ind./L) remaining dominant alongside high protozoan densities (4285.71 ind./L). Spring represented the peak in zooplankton abundance (19,048.55 ind./L), driven primarily by rotifers (13,000 ind./L) and protozoans (5857.14 ind./L). Notably, large‐bodied crustaceans (cladocerans and copepods) consistently exhibited low abundances across all seasons, with annual mean densities of only 274.74 and 402.11 ind./L, respectively. Cluster analysis based on Bray–Curtis similarity of zooplankton abundance data revealed clear compositional variation among samples (Figure S2). Rotifers and protozoans were the primary contributors to the zooplankton community, though their relative proportions varied across samples. Cladocerans were prominent at site SUL5, whereas copepods were prominent at sites AUL3 and AUL4.

#### 3.2.3 Diversity Indices

**FIGURE 5:**
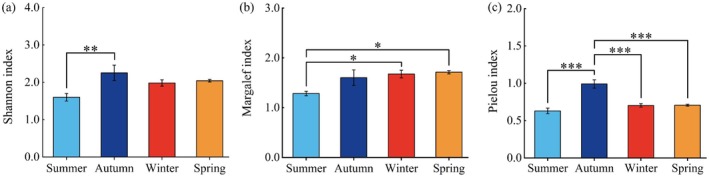
Seasonal patterns of zooplankton diversity indices in Changhu Lake. (a) Shannon index; (b) Margalef index, and (c) Pielou index. Error bars represent standard error. Asterisks indicate significant seasonal differences. *p < 0.05, **p < 0.01, ***p < 0.001.

Zooplankton community diversity indices exhibited pronounced seasonal dynamics (Figure 5). The Shannon‐Wiener index in autumn was significantly higher than that in summer (*p* < 0.01), while spring and winter showed comparable values (2.03 and 1.98, respectively), and summer had the lowest diversity (Figure 5a). The Margalef richness index also varied significantly (Figure 5b), peaking in spring (mean = 1.71), followed by winter (1.67) and autumn (1.60), and reaching its lowest point in summer (1.28). The Pielou evenness index was highest in autumn (0.99), differing significantly from other seasons (*p* < 0.001; Figure 5c). Winter (0.71) and spring (0.70) showed similar evenness, while summer was lowest (0.63). These fluctuations reflect dynamic shifts in community structure in response to environmental drivers.

Ecological quality assessment using these indices is summarized in Figure S3. The Shannon index indicated relatively low quality across all seasons (Figure S3a), with most sites classified as “Poor” in summer (85%) and autumn (67%). Conditions improved in winter and spring, with most sites rated “Medium”, though 5% of winter sites remained “Very poor”. The Margalef index showed similar temporal patterns (Figure S3b). Higher proportions of sites were “Poor” or “Very poor” in summer and autumn, with only 5% of autumn sites achieving “Medium”. Winter and spring showed better conditions, with “Medium” ratings for 24% and 15% of sites, respectively. In contrast, the Pielou index indicated consistently higher ecological quality (Figure S3c). Sites rated “Good” accounted for 65% and 43% in summer and autumn, respectively, with 24% of autumn sites “Excellent”. In winter and spring, the proportion of “Good” sites increased to 90%, with 5% “Excellent”. These findings highlight seasonal shifts in ecological integrity, with evenness metrics suggesting greater community stability compared to richness and diversity measures.

### 3.3 Community Stability

**FIGURE 6:**
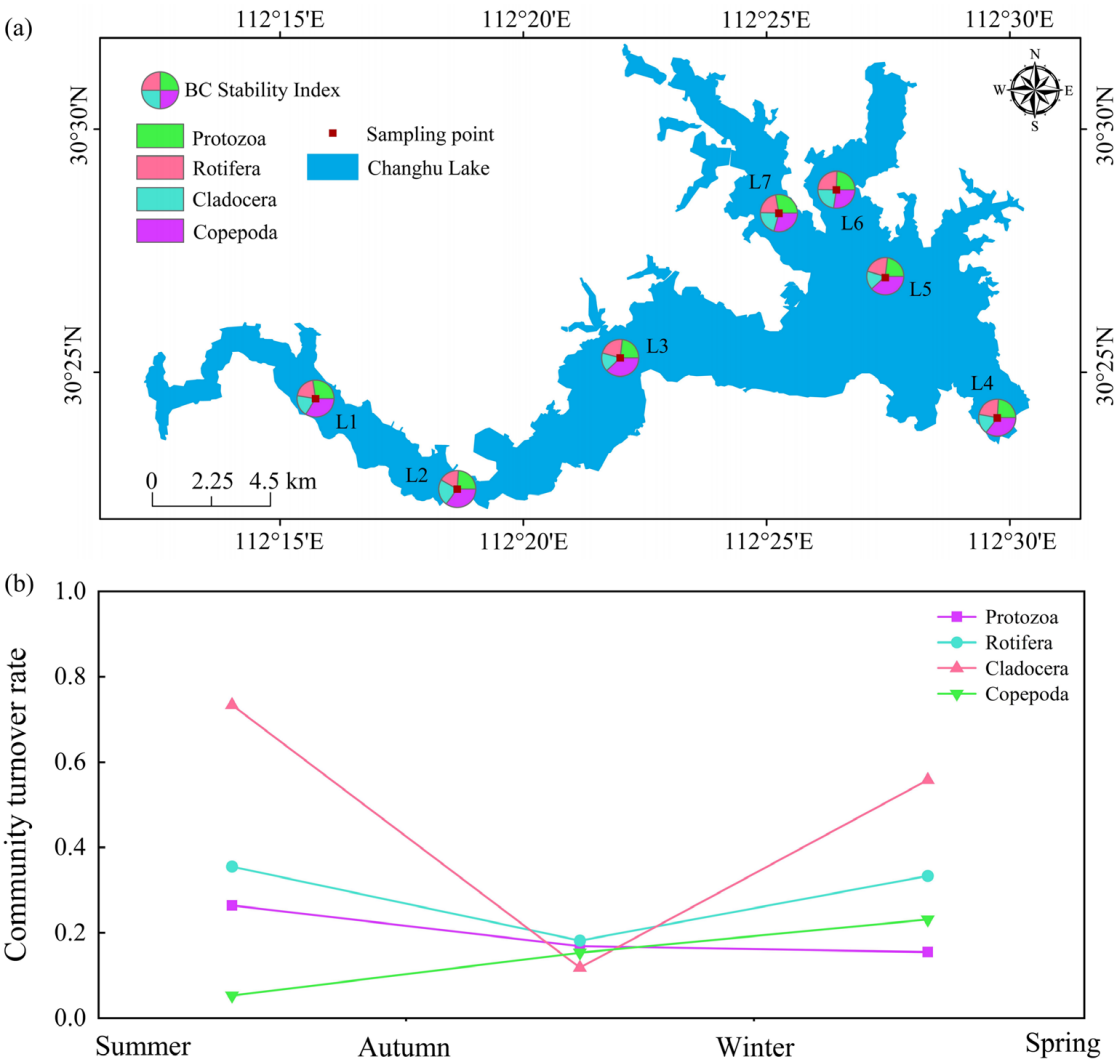
Zooplankton community stability in Changhu Lake. (a) Spatial variation of zooplankton stability index. Pie charts show the relative contribution of the stability index for zooplankton community (Protozoa, Rotifera, Cladocera, Copepoda). (b) Seasonal variation of zooplankton community turnover stability index.

The zooplankton community turnover stability index exhibited pronounced spatiotemporal heterogeneity (Figure 6a). Spatially, stability indices for protozoans, rotifers, and copepods exceeded 20% at all sites except L2. Cladocerans showed relatively lower stability (< 20%) at L1, L3, L4, and L5. In contrast, protozoans consistently demonstrated significantly greater stability than other groups lake‐wide, maintaining values above 30% at all sites.

Temporally, rotifer and cladoceran stability followed a biphasic pattern, declining initially then resurging, peaking during the summer‐winter transition (Figure 6b). Protozoan stability declined persistently throughout the sampling period, while copepods increased gradually, achieving the highest annual stability. Cladocerans reached maximum stability indices of 0.79 and 0.56 during the summer‐autumn and winter–spring transitions, respectively, significantly exceeding those of other taxa. Except for cladocerans, stability indices for all other groups fluctuated moderately around 0.2, indicating relatively low temporal variability.

### 3.4 Factors Influencing Community Stability

**FIGURE 7:**
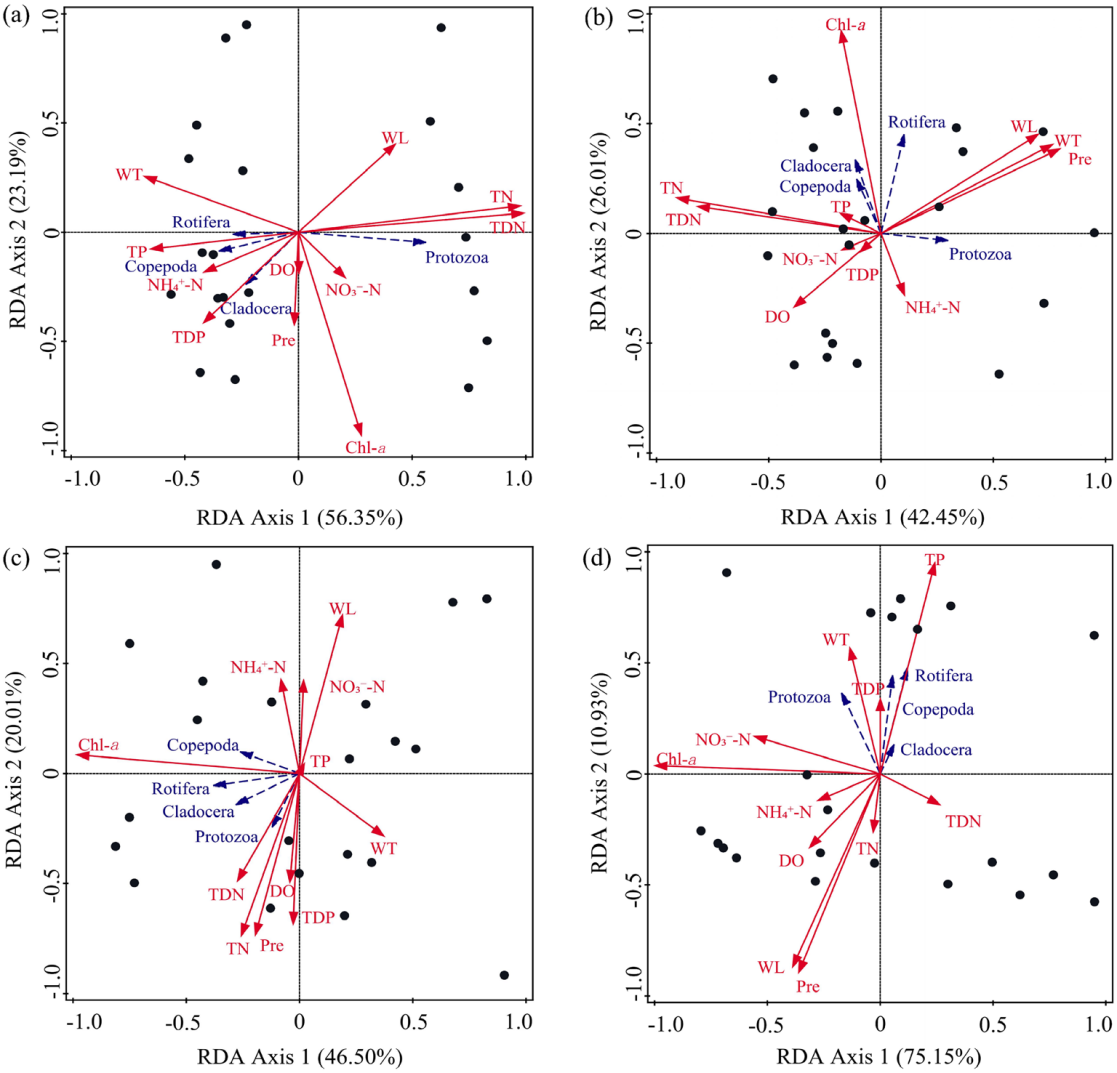
Redundancy analysis (RDA) of zooplankton communities and environmental variables across (a) summer, (b) autumn, (c) winter, (d) spring. Blue dashed arrows: zooplankton groups; red solid arrows: environmental variables. Black points represent sampling events.

RDA revealed distinct seasonal dynamics in the zooplankton‐environment relationship (Figure 7). In summer, the first two axes explained 79.54% of variation (Figure 7a). Water level (WL), TN, and TDN were negatively correlated with Rotifera, Cladocera, and Copepoda, but positively associated with Protozoa. Conversely, TP, precipitation, and Chl‐*a* were positively correlated with Rotifera, Cladocera, and Copepoda, but negatively correlated with Protozoa.

In autumn, the first two axes explained 68.46% of variance (Figure 7b). WL, WT, and precipitation positively correlated with Rotifera and Protozoa, while NH_4_^+^‐N was negatively associated with Cladocera and Copepoda. TN and TDN were negatively correlated with Protozoa, and Chl‐*a* was positively linked to Cladocera, Copepoda, and Rotifera.

In winter, the first two axes explained 66.51% of variation (Figure 7c). Chl‐*a* positively influenced the overall community. WL, NH_4_^+^‐N, and NO_3_^−^‐N correlated negatively with Cladocera and Protozoa but positively with Copepoda.

In spring, zooplankton assemblages were predominantly positively associated with WT, TDP, and TP, and negatively correlated with DO, WL, Pre, and TN (Figure 7d).

**FIGURE 8:**
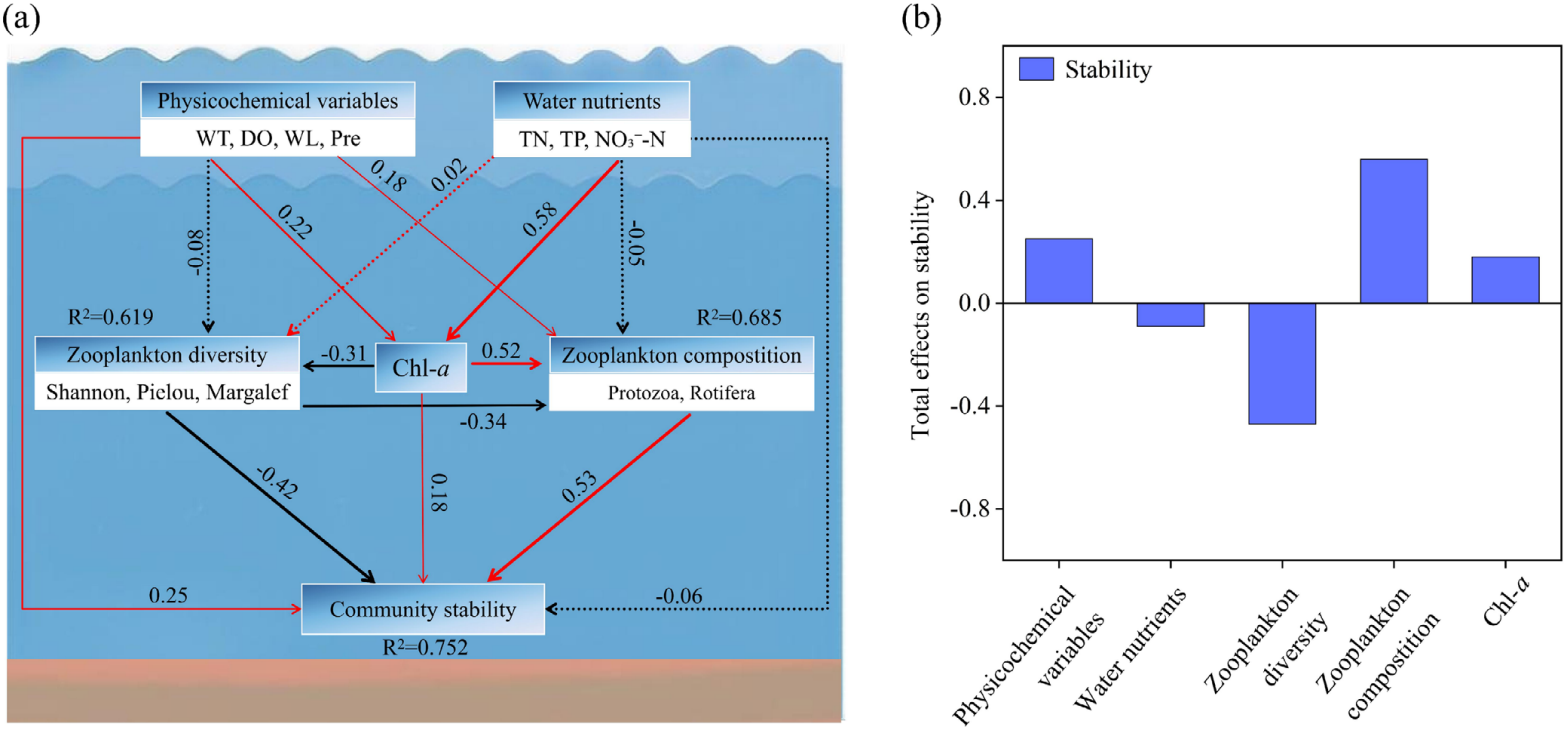
Driving mechanisms of zooplankton community stability. (a) Structural equation model (SEM) illustrating pathways through which physicochemical variables and nutrients affect diversity, composition, and stability. Red and black arrows indicate positive and negative effects, respectively. The numbers adjacent to the arrows are standardized path coefficients. Solid lines denote statistically significant paths (*p* < 0.05), and dotted lines represent non‐significant paths (*p* ≥ 0.05). Red lines represent positive correlations, and black lines represent negative correlations. *R* ^2^ values represent explained variance. (b) Total effects of environmental variables on stability.

The SEM delineated pathways through which abiotic factors regulate zooplankton community stability (Figure 8a). Community structure exerted the strongest positive direct effect on stability (path coefficient = 0.53). Diversity had a significant negative direct effect on stability (−0.42) and indirectly negatively affected stability via community structure (−0.34). Chlorophyll *a* (Chl‐*a*) had a positive effect on both community structure (0.52) and stability (0.18), but a negative effect on diversity (−0.31). Water physicochemical variables had a moderate positive direct effect on stability (0.25) and positively influenced community structure (0.18), but a minor negative effect on diversity (−0.08). Nutrient variables had a significant negative direct effect on Chl‐*a* (0.58), but a weak direct effect on stability (−0.06) and negligible paths to diversity (0.02) and community structure (−0.05), suggesting their impact is largely indirect.

Total effects analysis (Figure 8b) confirmed that community structure had the strongest overall positive influence on stability, followed by Chl‐*a* and water physicochemical variables. Diversity and nutrients showed negative total effects. These findings highlight that zooplankton stability in Changhu Lake is governed primarily by biotic interactions, with community structure as the central determinant, while nutrient enrichment may destabilize the system via multiple pathways.

### 3.5 Community Assembly

**FIGURE 9:**
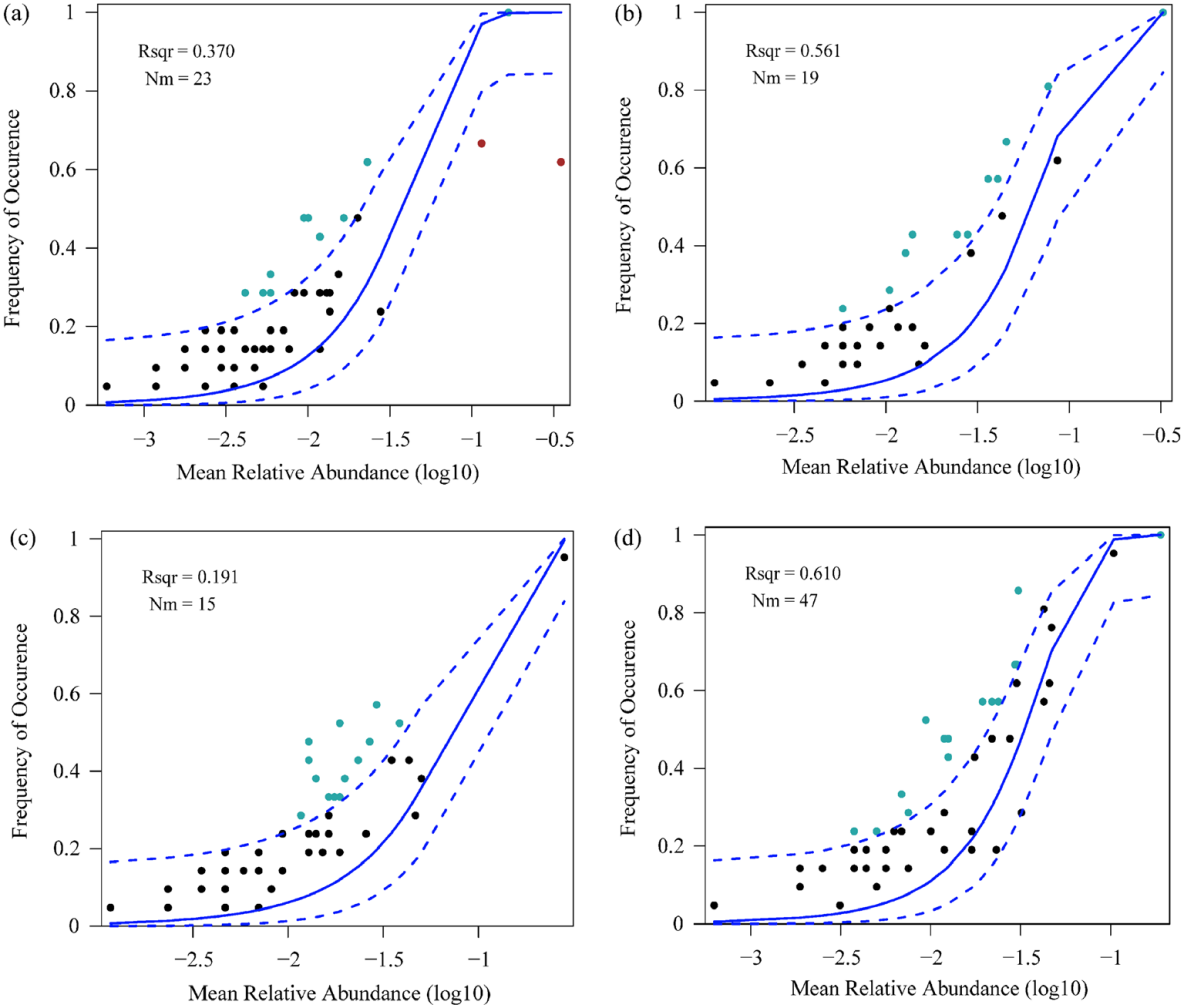
Neutral community model (NCM) fit for zooplankton assembly across seasons. (a) Summer, (b) autumn, (c) winter, (d) spring. Blue solid line: Predicted occurrence frequency; blue dashed lines: 95% confidence intervals. Black points fall within the confidence interval, turquoise above, and red below. *R* ^2^ represents goodness of fit; Nm represents the total mean immigration rate of zooplankton. The *y*‐axis (frequency of occurrence) corresponding to the cumulative distribution function of the Beta distribution.

The neutral community model (NCM) revealed marked seasonal variability in zooplankton assembly mechanisms (Figure 9). In spring, 23% of OTUs fell outside the 95% confidence intervals. Summer showed an outlier proportion (15%), autumn 20%, and winter 20%. The goodness‐of‐fit statistic (*R* ^2^) was highest in spring (0.610), followed by autumn (0.561), summer (0.370), and lowest in winter (0.191). While the low *R* ^2^ in winter might appear to indicate increased deterministic processes, the similar outlier proportions across seasons argue against a simple seasonal shift from stochastic to deterministic assembly. The immigration rate (Nm) was highest in spring (Nm = 47), followed by summer (23), autumn (19), and lowest in winter (15). This suggests a greater total mean migration rate in spring and stronger dispersal limitation in winter, underscoring that seasonal hydrological processes significantly influence zooplankton dispersal.

## 4 Discussion

### 4.1 Temporal and Spatial Variations of the Zooplankton Community

This study reveals that the zooplankton community in Changhu Lake displayed distinct seasonal dynamics in response to environmental changes, reflected in noticeable temporal heterogeneity in species composition, population density, and community structure (Rogers et al. 2020). The marked increase in rotifer abundance during summer was mainly driven by elevated water temperatures, intensified nutrient loading, and peak chlorophyll‐*a* concentrations (Greco et al. 2023). As opportunistic strategists with rapid reproductive rates and high tolerance to cyanobacterial blooms, rotifers thrived under eutrophic and warm conditions, becoming the dominant taxon in this season (Zhao et al. 2021; Mutethya et al. 2024). In contrast, protozoans maintained relatively stable and high abundances throughout the year, highlighting their functional significance in the zooplankton assemblage. Their ecological generality, broad trophic adaptability, and resilience to environmental fluctuations allow them to occupy foundational niches in the lake's food web (Li et al. 2022).

In autumn, despite a noticeable reduction in nutrient levels, diversity indices (e.g., Shannon‐Wiener and Margalef) increased significantly, and the community showed greater evenness and taxonomic balance. The shift toward moderate temperatures and improved water clarity supported more diverse phytoplankton assemblages, which in turn provided varied trophic resources and enhanced niche complementarity among zooplankton species (Zhao et al. 2022). This pattern aligns with the intermediate disturbance hypothesis, suggesting that autumn represents an ecologically transitional period with partial restructuring and strengthened resilience following the cessation of fishing (Labuce et al. 2021).

During winter, low water temperatures and reduced nutrient inputs favored stress‐tolerant taxa such as cold‐adapted rotifers (Wang et al. 2022; Boulianne et al. 2025). Concomitantly, diminished primary productivity limited the growth of other zooplankton groups, resulting in a simplified community structure with reduced trophic complexity. In spring, rising temperatures and increased nutrient availability stimulated phytoplankton growth, which likely prompted a synchronous increase in protozoan and rotifer populations. This reflects the community's capacity for seasonal recovery and dynamic adjustment under improved trophic conditions (Lomartire et al. 2021; Yi et al. 2024).

Spatially, relatively lower zooplankton diversity and higher abundance were recorded at southeastern sampling sites. This pattern is likely due to the region's geomorphological traits—low elevation, active hydrological connectivity, and enhanced water mixing—which facilitate allochthonous nutrient inputs and promote habitat heterogeneity (Zhang et al. 2019). These findings emphasize the importance of spatial processes and habitat filtering in shaping zooplankton communities under anthropogenic influence (Wu et al. 2022; dos Santos et al. 2025).

### 4.2 Relationship Between Zooplankton Community Structure and Stability

Conventional ecological theory suggests that higher biodiversity enhances ecosystem stability; however, observations from Changhu Lake indicate that this relationship may not hold during initial stages of ecosystem restructuring. Although Shannon diversity and Pielou's evenness indices peaked in autumn, ecosystem stability did not correspondingly increase and instead correlated negatively with diversity (Key et al. 2022; Kunze et al. 2025). This implies that increased diversity does not necessarily enhance stability in transitional systems (Filstrup et al. 2014; Qi et al. 2025). SEM further revealed that diversity indirectly reduced stability by adversely affecting community structure. This may reflect destabilizing mechanisms such as intensified interspecific competition and functional imbalance accompanying rapid diversity increases.

In contrast, community structure emerged as the strongest positive predictor of stability, underscoring the role of compositional integrity and functional complementarity in maintaining ecosystem homeostasis (Geng et al. 2022; Guo, Li, et al. 2025). Further analysis indicated that although large‐bodied crustaceans (e.g., cladocerans and copepods) were relatively scarce, they maintained high turnover stability during winter and spring. These taxa exhibit *K*‐selected life‐history strategies, characterized by persistence and stability under resource‐limited and stressful conditions (Song et al. 2020). In comparison, protozoan stability was more sensitive to nutrients and temperature fluctuations, displaying *r*‐selected traits such as rapid response, high plasticity, and lower temporal consistency (Calbet et al. 2022). These results emphasize that ecosystem stability is influenced not only by species richness but also by the life‐history strategies of dominant taxa and the interactive structure of the community.

RDA identified nutrient loading and hydrological conditions as the main abiotic drivers of community variation (Li, Lei, et al. 2025; Li, Pan, et al. 2025). In summer and autumn, total phosphorus and chlorophyll‐*a* showed strong positive correlations with rotifer, cladoceran, and copepod abundances, indicating that short‐term nutrient enrichment promotes opportunistic growth. Moreover, the varying effects of nitrogen forms (e.g., NO_3_^−^‐N vs. NH_4_^+^‐N) across seasons and taxonomic groups highlight the importance of nutrients in regulating zooplankton trophic dynamics and functional composition (Abd. Razak and Sharip 2019).

The SEM framework further clarified abiotic influence pathways (Li, Lei, et al. 2025; Li, Pan, et al. 2025). Physicochemical factors—especially dissolved oxygen, water temperature, and water level fluctuations—exerted direct effects on stability and indirectly supported resilience by optimizing community structure (Ahmed et al. 2025). Nutrient factors had weaker direct effects, operating mainly through structural mediation. Importantly, under continued eutrophication, excessive nutrient inputs may trigger compositional shifts, weaken species interactions, and disrupt community coherence, thereby reducing ecosystem stability (Vezi et al. 2019; He et al. 2021).

Additionally, hydrodynamic processes serve as key external modulators of zooplankton spatial heterogeneity and community turnover. By affecting habitat connectivity, nutrient diffusion, and phytoplankton dynamics, hydrodynamics regulates zooplankton dominance patterns and spatial restructuring. These findings collectively highlight the need for integrated watershed management strategies that consider hydrology, nutrient fluxes, and habitat heterogeneity to enhance ecosystem resilience and long‐term stability during restoration (Filstrup et al. 2014; Lomartire et al. 2021).

### 4.3 Differences in Zooplankton Community Assembly Mechanisms

Neutral community model (NCM) fitting results indicate significant seasonal variation in the apparent fit of the model to zooplankton communities in Changhu Lake. However, the proportion of outliers (OTUs falling outside the 95% confidence intervals) was similar across seasons. This suggests that neutral processes—i.e., demographic stochasticity and ecological drift—are insufficient to fully explain community assembly in any season, and that non‐neutral forces (e.g., environmental filtering, niche differentiation) operate throughout the year (Zhu et al. 2022). In terms of underlying mechanisms, spring communities exhibited relatively uniform physicochemical conditions and high nutrient availability. Under these circumstances, demographic stochasticity (random death and birth events) and ecological drift may play a more prominent role relative to other seasons, but deterministic filtering still affects a non‐negligible subset of taxa (Mao et al. 2025). In winter, increased abiotic stress (e.g., low temperatures, reduced productivity), likely strengthens niche‐based selection and favors specially adapted taxa (Valencia et al. 2004; Zheng et al. 2025). Nevertheless, the similar outlier proportions across seasons imply that the balance between stochastic and deterministic processes shifts only subtly, rather than switching entirely from one regime to the other.

Differential responses among zooplankton taxa further illustrate this mechanism: rotifers and protozoans consistently showed higher neutrality across seasons, likely due to their small size, short generation times, and high dispersal capacity (Qiu et al. 2025). In contrast, larger‐bodied cladocerans and copepods were more often identified as outliers, indicating higher sensitivity to deterministic filters such as environmental heterogeneity and resource partitioning (Alvarez et al. 2025; Qi et al. 2025). Furthermore, the observed seasonal patterns underscore the dynamic balance between stochastic and deterministic processes in aquatic communities. During periods of moderate disturbance and resource enrichment (e.g., spring and summer), demographic stochasticity and drift may have slightly greater relative importance, promoting assembly via random colonization and drift. Conversely, under greater habitat heterogeneity or physical stress (e.g., winter), niche‐based processes such as resource specialization and environmental filtering become more influential, as reflected by which taxa appear as outliers (Connell 1978; Bellier et al. 2022). This temporal pattern aligns with established successional frameworks observed in shallow, hydrologically connected lakes, where community assembly shifts in the relative balance between stochastic and deterministic regimes in response to fluctuating environmental gradients and biotic interactions (Zhang et al. 2022; Mutethya et al. 2024).

### 4.4 Ecological Responses and Management Implications

Despite the restoration initiatives implemented in Changhu Lake, monitoring during the study period revealed no significant recovery of large‐bodied zooplankton, nor abrupt changes in species richness or overall community composition. This suggests that early ecological responses were primarily expressed through enhanced system stability and increased environmental sensitivity of the zooplankton community, rather than through structural reorganization (Zhou et al. 2020). Such delayed responses are consistent with well‐established time‐lag effects commonly observed in the ecological restoration of shallow eutrophic lakes subject to long‐term anthropogenic pressure (Hu et al. 2022; dos Santos et al. 2025).

The immediate ecological effects of the restoration initiatives appear to involve dampened community fluctuations and improved environmental responsiveness. However, structural recovery of key functional groups—particularly cladocerans and copepods, which serve as crucial primary consumers in the pelagic food web—is likely to require an extended period (Yang et al. 2023; Guo, Wang, et al. 2025). Persistent non‐point source pollution and legacy nutrient loading continue to impair habitat quality, limiting the resurgence and functional reintegration of these taxa (Chen et al. 2025).

Comparative analysis with 2012–2013 data indicated an approximate 61% increase in total zooplankton abundance, accompanied by a marked shift in community composition toward Rotifer dominance (Figure S4). Notably, protozoan populations exhibited incomplete recovery, remaining at approximately 82% of their 2012–2013 levels. The observed community shift toward smaller‐bodied, *r*‐strategist taxa suggests an ecosystem still in an early transitional and reassembly phase. These changes likely result from the combined effects of persistent eutrophication, altered trophic dynamics, and residual environmental stressors. This pattern is consistent with recovery trajectories reported in other highly eutrophic shallow lakes (e.g., Lake Taihu, Lake Chaohu), where functional group reestablishment and structural reorganization typically occur only after 2–5 years of sustained management intervention (Zhou et al. 2020).

While restoration initiatives provide a crucial regulatory framework for ecosystem recovery, its long‐term ecological effectiveness depends on the implementation of integrated management strategies (Mills et al. 2023). These should include sustained ecological monitoring, targeted nutrient and pollution control, and restoration of habitat heterogeneity. Nevertheless, our study has several limitations. First, our sampling campaign may underestimate the abundance and diversity of micro‐organisms, as some individuals may pass through the mesh or be lost during the concentration and preservation processes (Thomas et al. 2017). Second, fish and phytoplankton sampling data are required to elucidate the detailed impacts of grazing and food pressure on zooplankton. Fish can exert strong top‐down control on zooplankton communities through selective predation, particularly on large‐bodied cladocerans and copepods, which can cascade through trophic networks and alter zooplankton community structure, size distribution, and stability (Jeppesen et al. 2011). Future monitoring should: (1) employ complementary techniques (e.g., high‐throughput sequencing, larger sample volumes, or finer mesh nets) to obtain a more complete assessment of zooplankton community composition in Changhu Lake; (2) integrate concurrent surveys of fish communities, phytoplankton composition, and zooplankton to fully understand the mechanisms driving zooplankton community stability in Changhu Lake, including the relative importance of top‐down versus bottom‐up forces across seasons; (3) prioritize dynamic indicators of community structure and function—especially diversity‐stability relationships and functional redundancy—to assess whether the system is approaching ecological thresholds or shifting toward alternative stable states (Du et al. 2024; Zhai et al. 2025). Such information is essential for scientifically evaluating the effectiveness of environmental protection policies and guiding adaptive management strategies aimed at accelerating ecological resilience and recovery.

## 5 Conclusions

This study systematically evaluates the zooplankton community structure, stability, and environmental responses in Changhu Lake. The results indicate that while some ecological attributes have shifted, the community has not yet undergone substantial structural reorganization, with protozoans and rotifers remaining dominant and large‐bodied taxa like cladocerans and copepods recovering only slowly. A notable negative correlation between diversity and stability was observed, suggesting that stability at this early restoration stage relies more on the structural and functional traits of specific taxa than on species richness. Total phosphorus, ammonium nitrogen, and Chlorophyll‐*a* were identified as key environmental drivers indirectly shaping community composition. Neutral community model fitting further revealed seasonal shifts in assembly mechanisms—stochastic processes prevailed in spring, whereas deterministic filtering dominated in winter. Although zooplankton stability improved, signaling enhanced buffering capacity, the limited recovery of ecologically pivotal groups highlights that the system remains in an early restorative phase. Future efforts should prioritize nutrient reduction, habitat enhancement, and targeted biotic interventions to promote structural reassembly and long‐term ecosystem resilience.

## Author Contributions

**Shihao Tang:** data curation (equal), investigation (equal), methodology (equal), writing – original draft (equal). **Jianqiang Zhu:** conceptualization (equal), funding acquisition (equal), supervision (equal). **Zilong Nie:** formal analysis (equal), investigation (equal), visualization (equal). **Jun R. Yang:** conceptualization (equal), funding acquisition (equal), validation (equal), writing – review and editing (equal).

## Funding

This work was supported by the National Natural Science Foundation of China (U21A2039 and 41901135) and the Jingzhou Science and Technology Plan Project (2025EB31).

## Conflicts of Interest

The authors declare no conflicts of interest.

## Supporting information


**Figure S1:** Temporal variation of precipitation and water level in Changhu Lake (June 2023–May 2024). (a) Monthly precipitation. (b) Monthly water level. Vertical dashed lines mark seasonal divisions.
**Figure S2:** Cluster analysis of zooplankton community composition based on Bray–Curtis dissimilarity among samples. SU, summer; AU, autumn; WI, winter; SP, spring.
**Figure S3:** Ecological quality assessment based on (a) Shannon‐Wiener index, (b) Margalef index, and (c) Pielou evenness index of the zooplankton community. Rings from outer to inner: summer, autumn, winter, spring. Color codes: Excellent (green), Good (orange), Medium (light purple), Poor (dark purple), Very poor (cyan).
**Figure S4:** Comparison of zooplankton abundance during 2012–2013 and 2023–2024 in Changhu Lake.
**Table S1:** Diversity index score table.

## Data Availability

All data supporting the findings of this study are publicly available in the Science Data Bank at https://www.scidb.cn/en/s/e6rQza.
