# Supplementary material for: Zooplankton Community Stability and Environmental Filtering in a Shallow Eutrophic Lake
Source: Ecol Evol. 2026 May 31;16(6):e73773. doi: 10.1002/ece3.73773 (PMC13239300; doi:10.1002/ece3.73773)
Supplement: Supplementary file 1 — Figure S1: Temporal variation of precipitation and water level in Changhu Lake (June 2023–May 2024). (a) Monthly precipitation. (b) Monthly water level. Vertical dashed lines mark seasonal divisions. Figure S2: Cluster analysis of zooplankton community composition based on Bray–Curtis dissimilarity among samples. SU, summer; AU, autumn; WI, winter; SP, spring. Figure S3: Ecological quality assessment based on (a) Shannon‐Wiener index, (b) Margalef index, and (c) Pielou evenness index of the zooplankton community. Rings from outer to inner: summer, autumn, winter, spring. Color codes: Excellent (green), Good (orange), Medium (light purple), Poor (dark purple), Very poor (cyan). Figure S4: Comparison of zooplankton abundance during 2012–2013 and 2023–2024 in Changhu Lake. Table S1: Diversity index score table. [file ECE3-16-e73773-s001.doc]

*Supporting information of the article:*

**Zooplankton Community Stability and Environmental Filtering in a Shallow Eutrophic Lake**

Shihao Tang, Jianqiang Zhu, Zilong Nie, Jun R. Yang*

*MARA Key Laboratory of Sustainable Crop Production in the Middle Reaches of the Yangtze River (Co-construction by Ministry and Province)/Hubei Key Laboratory of Waterlogging Disaster and Agricultural Use of Wetland, College of Agriculture, Yangtze University, Jingzhou 434025, China*

* Corresponding author. E-mail: junyang2@yangtzeu.edu.cn (Jun R. Yang)

**This supplementary information contains:**

● Number of Pages: 6

● Number of Figures: 4

● Number of Table: 1


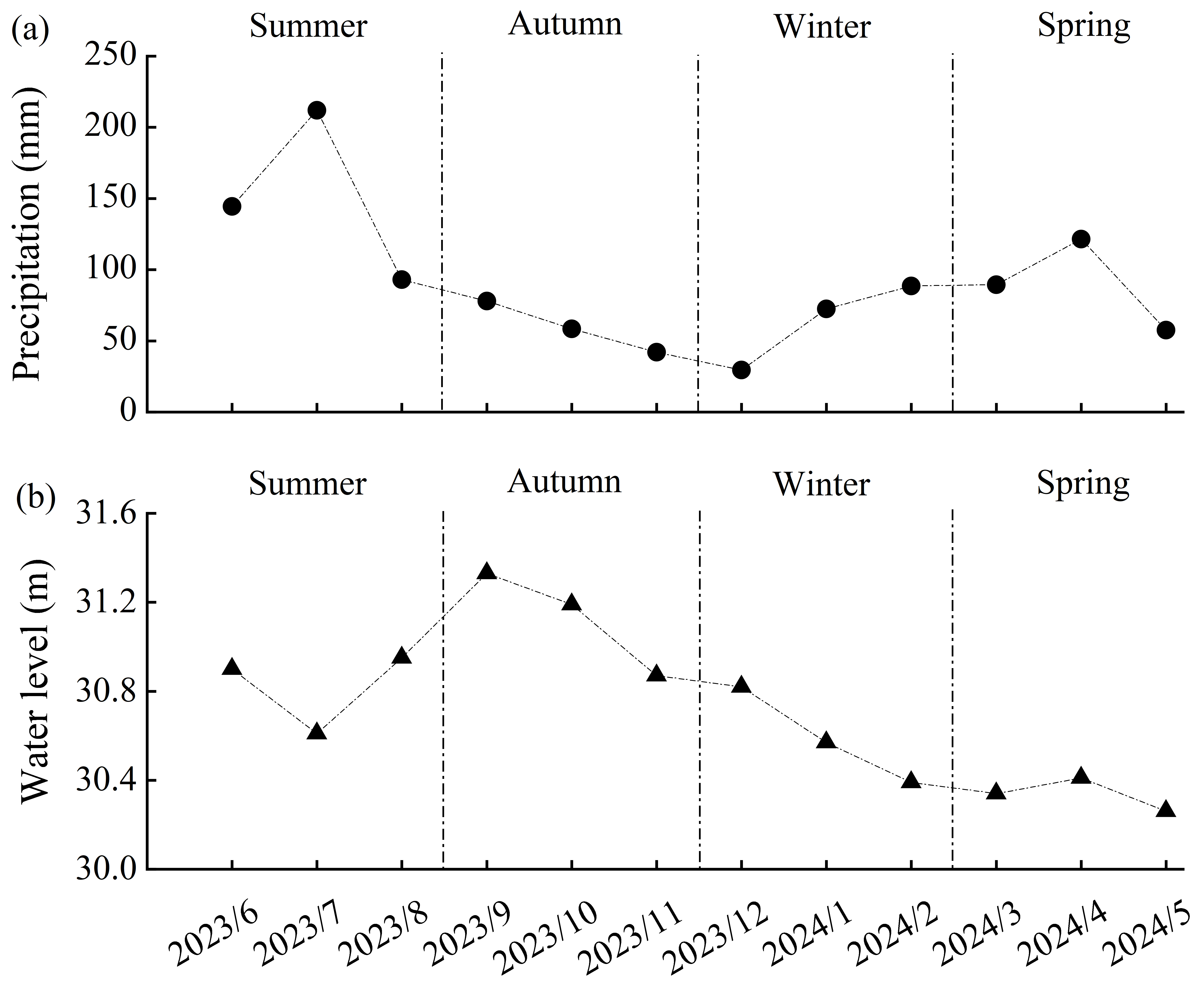


**FIGURE S1** Temporal variation of precipitation and water level in Changhu Lake (June 2023 - May 2024). (a) Monthly precipitation. (b) Monthly water level. Vertical dashed lines mark seasonal divisions.


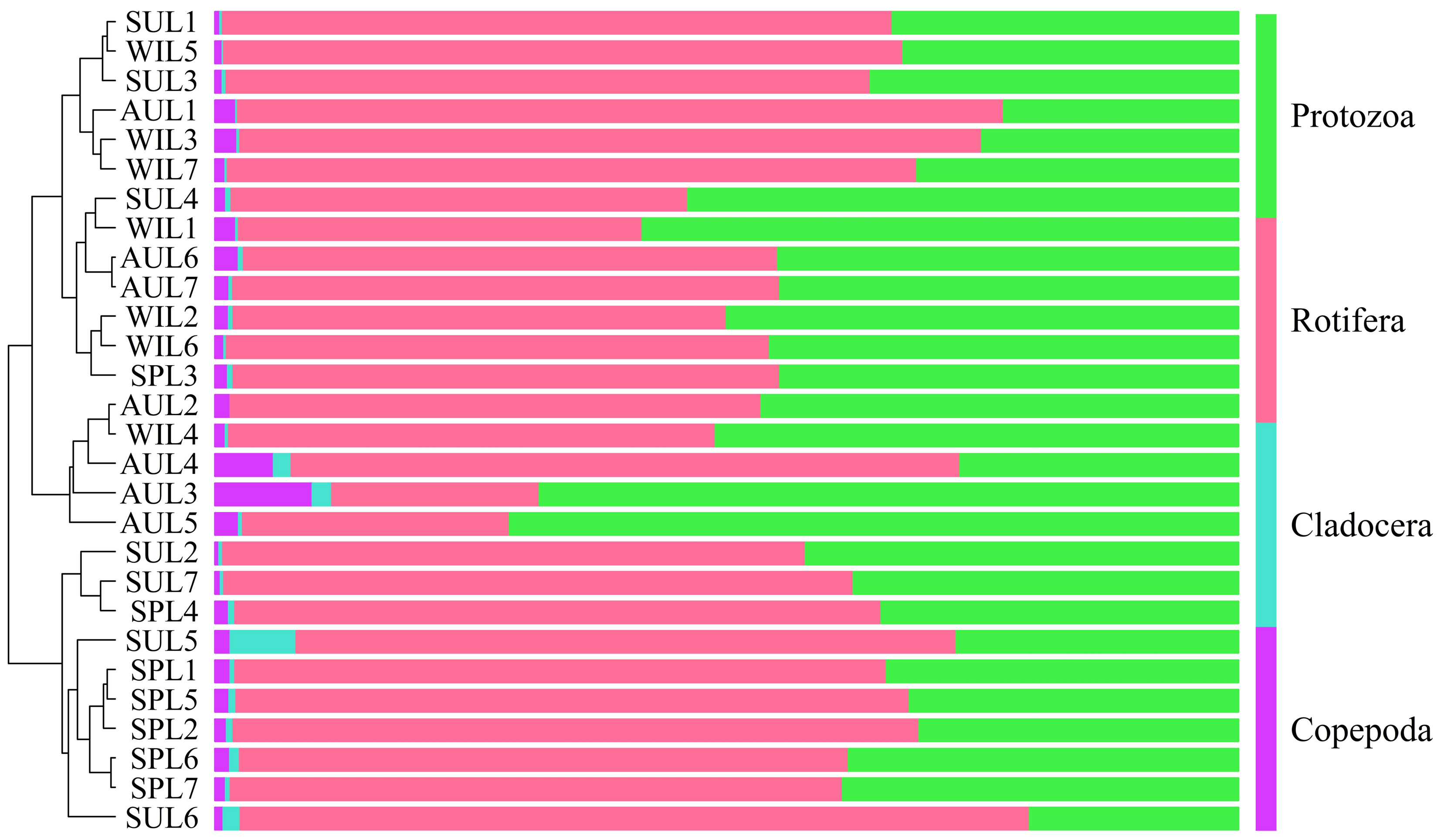


**FIGURE S2** Cluster analysis of zooplankton community composition based on Bray-Curtis dissimilarity among samples. SU, summer; AU, autumn; WI, winter; SP, spring.


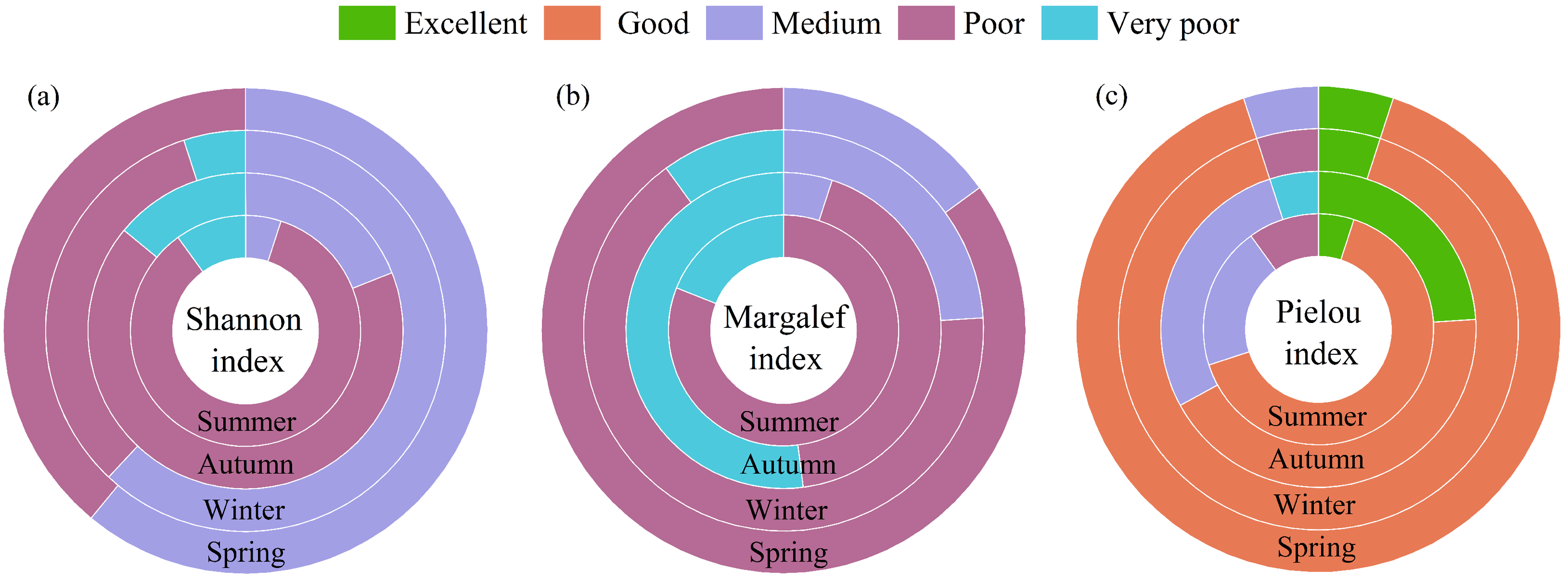


**FIGURE S3** Ecological quality assessment based on (a) Shannon-Wiener index, (b) Margalef index, and (c) Pielou evenness index of the zooplankton community. Rings from outer to inner: summer, autumn, winter, spring. Color codes: Excellent (green), Good (orange), Medium (light purple), Poor (dark purple), Very poor (cyan).


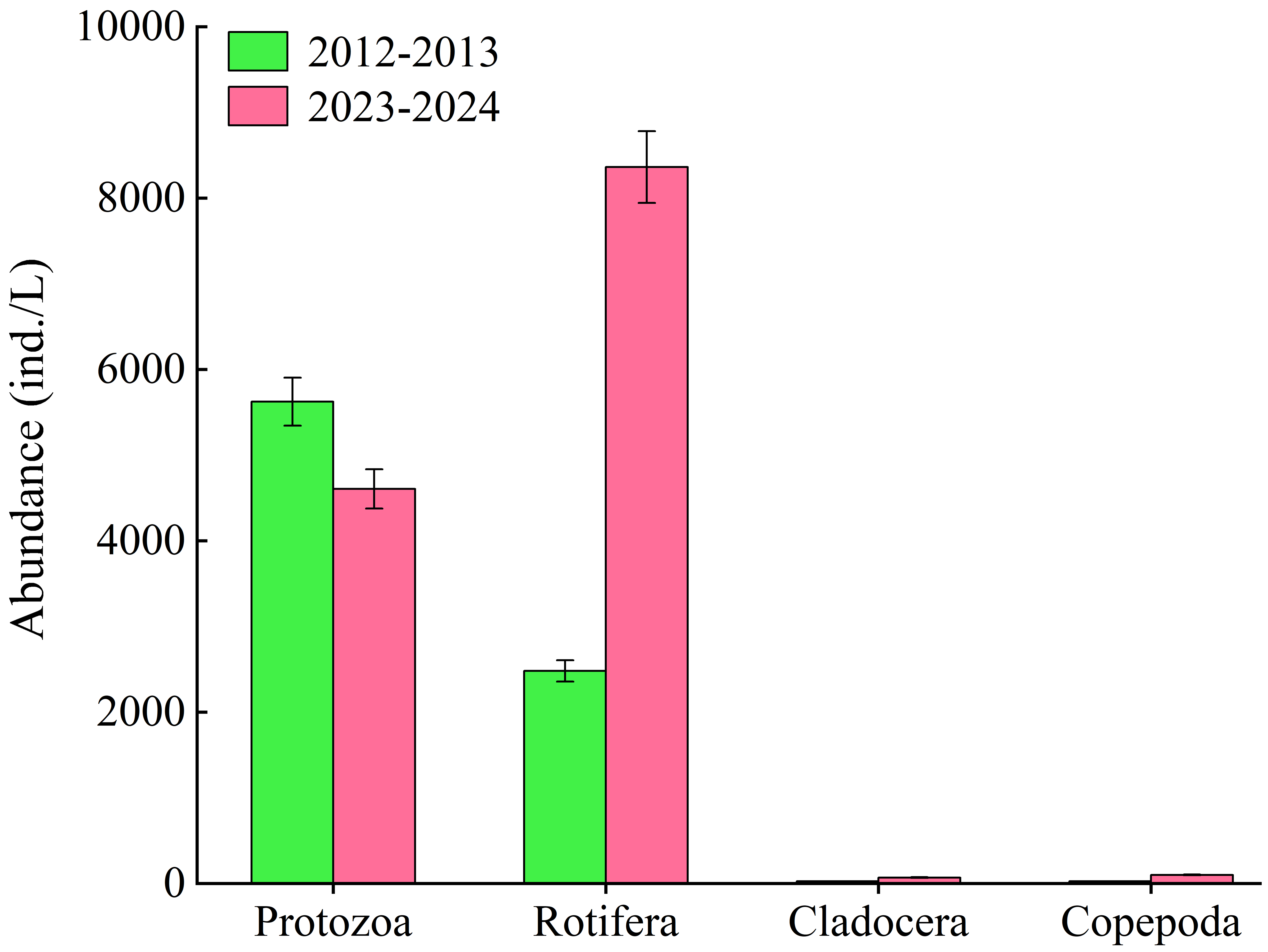


**FIGURE S4** Comparison of zooplankton abundance during 2012-2013 and 2023-2024 in Changhu Lake.

**TABLE S1** Diversity index score table.

|  | Very poor | Poor | Medium | Good | Excellent |
| --- | --- | --- | --- | --- | --- |
| *H′* | *H′* < 1 | 1 ≤ *H′* < 2 | 2 ≤ *H′* < 3 | 3 ≤ *H′* < 4 | *H′* ≥ 4 |
| *D* | *D* < 1 | 1 ≤ *D* < 2 | 2 ≤ *D* < 3 | 3 ≤ *D* < 4 | *D* ≥ 4 |
| *J* | *J* < 0.2 | 0.2 ≤ *J* < 0.4 | 0.4 ≤ *J* < 0.6 | 0.6 ≤ *J* < 0.8 | *J* ≥ 0.8 |
